# Multilevel logistic regression analysis of factors associated with delivery care service utilization among childbearing women in Ethiopia

**DOI:** 10.3389/frph.2023.1045964

**Published:** 2023-06-21

**Authors:** Naod Gebrekrstos Zeru, Dechasa Bedada Tolessa, Jaleta Abdisa Fufa, Bonsa Girma Fufa

**Affiliations:** ^1^ Department of Statistics, Mekelle University, Mekelle, Ethiopia; ^2^ Department of Statistics, Ambo University, Ambo, Ethiopia; ^3^ Department of Statistics, Jimma University, Jimma, Ethiopia; ^4^ Department of Statistics, Diredawa University, Diredawa, Ethiopia

**Keywords:** delivery skilled assistance, ANC visits, maternal health, multilevel model, delivery care services

## Abstract

Delivery service utilization is one of the key and proven interventions to reduce maternal death during childbearing. In Ethiopia, the utilization of health facilities for delivery service is still at a lower level. This study intends to model the determinant factors for the delivery care service utilization of childbearing mothers in Ethiopia using the 2016 Ethiopian demographic and health survey data. A cross-sectional study design was selected to assess factors associated with delivery care among mothers who had at least one child in the last 5 years before the survey aged 15–49 years in the data. Among these eligible mothers, 3,052 (27.7%) mothers had received delivery service care from health professionals. The results of multilevel logistic regression indicated that those at age 35–49 years (AOR = 0.7808, 95% CI: 0.5965–1.1132), an urban place of residence (AOR = 5.849 95% CI: 4.2755–8.0021), woman's higher level of education (AOR = 3.484, 95% CI: 2.0214–6.0038) and partner's higher educational level (AOR = 1.9335, 95% CI: 3,808–2.07352), household wealth index (AOR = 1.99, 95% CI: 1.724–2.3122), most every day exposed to mass media (AOR = 3.068, 95% CI: 1.456–6.4624), 2–4 birth order number (AOR = 0.604, 95% CI: 0.51845–1.4213), using contraceptive type (AOR = 1.4584, 95% CI: 1.2591–1.6249) and visiting more than 4 antenatal care visits (AOR = 7.574, 95% CI: 6.4824–8.84896) were more likely to give birth at a health facility compared to their counterparts. The woman's and partner's educational level, household wealth index, exposure to mass media and number of antenatal care visits had a positive association with delivery assistance whereas birth order had a negative association. The findings of this study were valuable implications to support strategies and interventions to address delivery care service in Ethiopia.

## 1. Introduction

Delivery care service is a service provided to mothers during labor, delivery, and the early postpartum period by accredited health professionals who have been educated and trained to proficiency in the skills needed to manage complications in women and newborns (1). Globally, The WHO estimates that more than 300,000 women died from pregnancy-related causes in 2015, which equates to 830 women every day. Of these, two-thirds (201,000) occurred in Sub-Saharan Africa, and about 22% (66,000) occurred in South Asia. Maternal mortality ratios are 29 times higher in low-income countries than in high-income countries (2).

The availability of delivery care services varies across the world. Globally, the Proportion of births attended by skilled health personnel is about 81%. In developed countries, the WHO estimates skilled attendance has reached 99%, while in Africa, the proportion of deliveries attended by skilled health personnel is only 59% (2). A study in 29 countries in sub-Saharan Africa used data from Demographic and Health Surveys (DHS) conducted between 1990 and 2015 showed that almost 40% of births are not attended by skilled personnel (3).

In Ethiopia, maternal deaths represent 25 percent of all deaths among women aged 15–49, and the current maternal mortality ratio is 353 per 100,000 live births. The aim of SDG By 2030 is to reduce the global maternal mortality ratio to less than 70 per 100 000 live births (2). The maternal death rate is mostly attributed to poor utilization of institutional delivery services. The proportion of births that occur at home remains higher, and the rate of births attended by skilled health professionals is very low. According to the Ethiopian Demographic and Health Survey (EDHS) 2016, only 26% of the births that year were delivered at a health facility (4).

A single-level analysis at the regional level cannot be used to make assumptions at the individual level. Individuals are nested within regions, and they tend to share certain common values. Thus, a woman receiving a delivery service is not only a result of her individual characteristics but also the result of some regional-level variables. The data used stem from a two-stage sampling design, wherein the first stage regions are sampled, and then a random sample of women was taken. Using a two-level model rather than a standard single-level regression had a big advantage in that it is possible to differentiate between several effects in a single-level model. With multilevel models, it is also possible to deal with complex variation at different levels it does not treat individuals or regions as having the same error variance. Therefore, this study intended to spot the determinant factors affecting delivery care service utilization of childbearing women in Ethiopia by considering heterogeneity in receiving a delivery service within the regions.

## 2. Materials and methods

The study was based on the EDHS conducted in 2016. The EDHS collects nationally representative data on women of child-bearing age (15–49 years) and their children. The 2016 EDHS sample is stratified and was selected in two stages. Each region was stratified into urban and rural areas, which yielded 21 sampling strata. Samples of EAs were selected independently in each stratum in two stages. In the 2016 EDHS, a representative sample of approximately 17,067 households from 645 clusters was selected. The sample was selected in two stages. In the first stage, 645 clusters (202 EAs in urban areas and 443 EAs in rural areas)) were selected from the list of Enumeration Areas (EA). In the interviewed households, 16,583 eligible women were identified for individual interviews; interviews were completed with 15,683 women, yielding a response rate of 95 percent. In the survey, information on delivery care was collected from women who had at least one birth in the 5 years before the survey. There was a toal of 10,641 cases from 643 clusters included in the analysis (4).

This study used assistance during delivery, which is defined in the EDHS 2016 report as whether the mothers received assistance from a certified health professional (doctor, nurse, midwife, health officer, and health extension worker).

So, the response variable of the *i*^th^ woman's was *Y_i_* measured as:

(1) $$Y_{i}=\{\begin{array}{cc} 1, & \;\mathrm{if}\;i^{\mathrm{th}}\;\mathrm{woman}\;\mathrm{get}\;\mathrm{services}\;\mathrm{from}\;\mathrm{any}\;\mathrm{health}\;\mathrm{professionals}\\ 0, & \mathrm{otherwise}\; \end{array}.$$

The variables in this study were independent variables which include demographic factors, socio-cultural factors, and economic factors. Independent factors that are expected to impact delivery care service utilization are age at birth, region, residence, woman's educational level, partner's educational level, occupation, religion, wealth, sex HH header, mass media, birth order, contraceptive use, and ANC visits.

### 2.1. Multilevel model analysis

Multilevel analysis is a methodology for the analysis of data manifesting complex variability, with a focus on nested sources of variability. The 2016 EDHS data set used for this study is based on multistage stratified cluster sampling. The appropriate approach to analyzing delivery care service data from this survey is therefore based on nested sources of variability. Here the units at the lower level are individual women who are nested within units that represent regions. Due to this nested structure, the odds of a woman receiving service during delivery are dependent, because women from the same region may share common exposure to the outcome of interest. In this study, the researcher considers a two-level hierarchical analysis where women are nested within regions.

### 2.2. Two level model

In this study, the clustering of the data points within geographical regions offers a natural 2-level hierarchical structure of the data, i.e., women are nested within regions. Let *y_ij_* be the binary outcome variable for individual woman *i* in region *j*, coded “0” or “1”, associated with level-one unit *i* nested within level-two unit *j*. Also let *p_ij_* be the probability that the response variable for individual *i* in region *j* equals 1, and *p_ij_* = Pr (*y_ij_* = 1). Here, *y_ij_* follows a Bernoulli distribution. Like logistic regression, the *p_ij_* is modeled using the link function, logit. The two-level logistic regression model can be written as,

(2) $$\mathrm{logit}(\;p_{ij})=\log\left[\frac{\;p_{ij}}{1-p_{ij}}\right]=\beta_{0}+\beta_{1}x_{ij}+U_{0j}$$

Where $u_{\;j0}$ is the random effect at level 2.

### 2.3. The empty logistic regression model

The empty two-level model for a dichotomous outcome variable refers to a population of groups (level-two units) and specifies the probability distribution for group-dependent probabilities $p_{j}$ in $Y_{ij}=p_{j}+\varepsilon_{ij\;}$ without taking further explanatory variables into account. This focuses on the model that specifies the transformed probabilities $f(\;p_{j})$ to have a normal distribution. This is expressed for a general link function f(p), by the formula;

(3) $$f(\;p_{j})=\beta_{0}+U_{0j}$$

Where, $\beta_{0}$ is the population average of the transformed probabilities and $U_{0j}$ is the random deviation from this average for group *j*.

### 2.4. The random intercept logistic regression model

In the random intercept model, the intercept is the only random effect meaning that the groups differ with respect to the average value of the response variable, but the relationship between explanatory and response variables cannot differ between groups. We assume that there are variables that potentially explain the observed success and failure. These variables are denoted by $X_{h},\;(h=1,2,\ldots ,k)$ with their values indicated by $X_{hij}$. Since some or all of those variables could be level one variables, the success probability is not necessarily the same for all individuals in a given group (5). Therefore, the success probability depends on the individual as well as the group, and is denoted by $P_{ij}$. The outcome variable is split into an expected value and residual as: $Y_{ij}=P_{ij}+\varepsilon_{ij\;}$.

The random intercept model expresses the log-odds, i.e., the logit of *P_ij_*, as a sum of a linear function of the explanatory variables. That is,

(4) $$\mathrm{logit}(P_{ij})=\log\left(\frac{\;p_{ij}}{1-p_{ij}}\right)=\beta_{0j}+\beta_{1}x_{1ij}+\beta_{2}x_{2ij}+\ldots +\beta_{k}x_{kij}=\beta_{0j}+\sum_{h=1}^{k}\beta_{h}x_{hij}$$

Where the intercept term $\beta_{0j}$ is assumed to vary randomly and is given by the sum of an average intercept $\beta_{0}$ and group-dependent deviations $U_{0j}$, that is

(5) $$\beta_{0j}=\beta_{0}+U_{0j}$$

As a result we have:

(6) $$\mathrm{logit}(P_{ij})=\beta_{0}+\sum_{h=1}^{k}\beta_{h}X_{hij}+U_{0j}\;$$

Where $\beta_{0}+\sum_{h=1}^{k}\beta_{h}X_{hij}$, is the fixed part of the model*.* The remaining *U*_0*j*_ is called the random part of the model. It is assumed that the residual *U*_0*j*_ are mutually independent and normally distributed with mean zero and variance $\sigma_{0}^{2}$.

### 2.5. Random coefficient logistic regression model

In logistic regression analysis, linear models are constructed for the log-odds. The multilevel analogue, random coefficient logistic regression is based on linear models for the log-odds that include random effects for the groups or other higher-level units.

Consider explanatory variables which are potential explanations for the observed outcomes. Denote these variables by $X_{1},X_{2},\ldots ,X_{k}$. The values of $X_{h}(h=1,2,\ldots ,k)$ are indicated in the usual way by *X_hij_*.

Now consider a model with group-specific regressions of logit of the success probability, $\mathrm{logit}(P_{ij})$, on a single level one explanatory variable *X*,

(7) $$\mathrm{logit}(P_{ij})=\log\left(\frac{\;p_{ij}}{1-p_{ij}}\right)=\beta_{0j}+\beta_{1j}x_{1ij}$$

The intercepts $\beta_{0j}$ as well as the regression coefficients or slopes, $\beta_{1j}$ are group dependent. These group dependent coefficients can be split into an average coefficient and the group dependent deviation:

(8) $$\beta_{0j}=\beta_{0}+U_{0j}$$

(9) $$\beta_{1j}=\beta_{1}+U_{1j}$$

Substitution into equation (3.22) leads to the model

(10) $$\mathrm{logit}(P_{ij})=\log\left(\frac{\;p_{ij}}{1-p_{ij}}\right)=(\beta_{0}+U_{0j})+(\beta_{1}+U_{1j})x_{1ij}\;=\beta_{0}+\beta_{1}x_{1ij}+U_{0j}+U_{1j}x_{1ij}$$

There are two random group effects, the random intercept *U*_0*j*_ and the random slope *U*_1*j*_. It is assumed that the level two residuals *U*_0*j*_ and *U*_1*j*_ has both zero mean given the value of the explanatory variable X. Thus, $\beta_{1}$ is the average regression coefficient and $\beta_{0}$ is the average intercept. The first part $\beta_{0}+\beta_{1}x_{1ij}$ is called the fixed part of the model whereas the second part $U_{0}+U_{1j}x_{1ij}$ is called the random part of the model.

The most common methods for estimating multilevel logistic models are based on likelihood. Among the methods, Marginal Quasi Likelihood or MQL and Penalized Quasi Likelihood or PQL are the two prevailing approximation procedures. Both MQL and PQL are based on Taylor series expansion to achieve the approximation. The model selection method in multilevel is based on likelihood ratio test and AIC.

## 3. Results

This study focused on a sample of 11,023 mothers from nine regional states and two city administrations in Ethiopia for the most recent birth within the 5 years preceding the EDHS 2016. The dataset was weighted to provide national estimates. Among the eligible mothers, 3,057 (27.7%) mothers received delivery care service from health professionals. The service rate is slightly higher (32.8%) for the 15–19 age group than the 20–34 age group (27.8%). The delivery service rate was lower (23.1%) for the 35–49 age group compared to the other age groups. The region with the lowest service rate during delivery was Afar (16.4%) followed by Oromia (19.7%) and the highest was Addis Ababa (96.7%) followed by Tigray (59.2%). The proportion of urban mothers who received delivery service was 80.1% and in rural areas the rate was only 21.2% (Table 1).

**Table 1:** Summary of descriptive statistics for delivery care service use.

Variables	Labels	Delivery assistance service use		Total
		No	Yes	
Age at birth	15–19	879 (67.2)	429 (32.8)	1,308
	20–34	5,890 (72.1)	2,200 (27.8)	8,090
	35–49	1,255 (76.9)	377 (23.1)	1,632
Region	Tigray	292 (40.8)	424 (59.2)	716
	Afar	96 (83.5)	19 (16.4)	114
	Amhara	1,497 (72.2)	575 (27.8)	2,072
	Oromia	3,895 (80.2)	956 (19.8)	4,851
	Somali	406 (79.9)	102 (20.1)	508
	Begshangul	87 (71.3)	35 (28.7)	112
	SNNP	1,640 (71.4)	656 (28.6)	2,296
	Gambela	14 (51.9)	13 (48.1)	27
	Harari	13 (50)	13 (50)	26
	AA	8 (3.3)	236 (96.7)	244
	Dire Dawa	20 (42.6)	27 (57.4)	47
Religion	Orthodox	2,350 (62.3)	1,422 (37.7)	3,772
	Protestant	1,788 (73.5)	6,455 (26.5)	2,433
	Muslim	3,590 (78.7)	971 (21.3)	4,561
	Others	242 (94.2)	15 (5.8)	257
Residence	Urban	242 (19.9)	973 (80.1)	1,216
	Rural	7,728 (78.8)	2,079 (21.2)	9,426
Woman education	No education	6,029 (82.8)	1,255 (17.2)	7,284
	Primary	1,811 (61.4)	1,140 (36.6)	2,951
	secondary	111 (21.6)	403 (78.4)	514
	higher	19 (6.9)	255 (93.1)	274
Partner's/husband's education	No education	4,092 (81.8)	911 (18.2)	5,003
	Primary	3,073 (74.7)	1,043 (25.3)	4,116
	Secondary	303 (38.0)	495 (62.0)	798
	Higher	143 (26.2)	402 (73.8)	505
Sex of HH	Male	6,874 (73.8)	2,445 (26.2)	9,319
	Female	737 (64.5)	406 (35.5)	1,143
Occupation	No working	4,426 (70.1)	1,464 (29.9)	5,890
	Agricultural	817 (59.7)	551 (40.3)	1,368
	HH and domestic	2,140 (78.7)	578 (21.3)	2,718
	Sales	236 (53.3)	207 (46.7)	443
	Service	283 (63.9)	160 (36.1)	443
	Others	18 (16.5)	91 (83.5)	109
Wealth index	Poor	4,026 (80.9)	948 (19.1)	4,974
	Middle	1,533 (68.4)	709 (31.6)	2,242
	Rich	2,411 (63.3)	1,396 (36.7)	3,807
Media exposure	Not at all	5,821 (80.5)	1,412 (19.5	7,233
	Less than a week	1,680 (66.8)	835 (32.2)	2,515
	At least a week	455 (42.3)	621 (57.7)	1,076
	Almost everyday	8 (4.2)	181 (95.8)	189
Birth order	First	1,033 (50.2)	1,026 (49.8)	2,059
	2–4	3,378 (71.6)	1,340 (28.4)	4,718
	5 and above	3,559 (83.8)	687 (16.2)	4,246
Contraceptive use	No	5,892 (78.2)	1,647 (21.8)	7,539
	Yes	2,079 (59.6)	1,405 (40.4)	3,484
No. of ANC visits	No visits	2,561 (90.3)	274 (9.7)	2,835
	1–3	1,507 (64.3)	837 (35.7)	2,344
	4 and above	1,009(41.8)	1,406(58.2)	2,415
Total		7,971(72.3)	3,052(27.7)	11,023

Table 1 showed that the delivery service proportion for non-educated mothers was 17.2%, for primary educated mothers 36.6%, for mothers with secondary education level 78.4%, and for above secondary educational level 93.1%. Regarding the educational level of their partners, the delivery service proportion for women whose partners are not educated was 18.2%, for mothers with primary educated partners 25.3%, and for mothers with secondary educated partners 62%.

### 3.1. Multilevel logistic regression analyses

A two-level structure (with individual women as the first-level unit and region as the second-level unit) has been used. For the proper application of multilevel analysis in multilevel logistic analysis in particular, the first logical step is to test for heterogeneity of proportions between regions. The chi-square test was applied to assess heterogeneity between regions means. The test results are *χ*^2^ = 1,859.2, *df* = 10, *p*-value (*p* < 0.0001). Thus, there is evidence of heterogeneity with respect to the reception of delivery service among women in regions of Ethiopia.

### 3.2. Analysis of the empty model with random intercept

The empty two-level model also called the null two-level model for a dichotomous outcome variable refers to a population of groups and specifies the probability distribution for group-dependent probabilities, 휋_푗_. It is the model that incorporates only the grand mean and random intercept (regional effect) without covariate.

The fixed part of the random intercept model in Table 2 can be interpreted as a grand mean of log odds of receiving a service with the odds of and the average probability of getting a delivery care service $\frac{\exp(0.658)}{1+\exp(0.658)}=0.397$ which means that on average the chance of receiving delivery service was 39.7%. In addition, Table 2 indicates that the random part which is the between-region variance is 1.82.

**Table 2:** Estimates for random intercept model only.

Fixed effects:						
Delivery	Coef.	Std. err.	*Z*	*P *>* *|*z*	[95% Conf. interval]	
Cons	−0.418	0.101	−4.16	<0.0001*	−0.6157	−0.2204
Random effects: region						
var(cons)	1.82	0.45		0.9225	2.2046	
ICC	0.461					
Deviance = 1,170, AIC = 11,711, BIC = 11,726						
Deviance-based residual = 1,839						

*Significant variable.

At the bottom of Table 2 the deviance value for this model is 11,707, and the deviance for the empty model without random effect is 13,546. This implies the deviance-based chi-square is 919.5 (13,546–11,707). This value is compared to chi-square distribution with 1 degree of freedom. The significance of it (*X*^2 ^= 919.5, *p*-value <0.0001) implies there is evidence of heterogeneity or cross-regional variation in the reception of delivery service. And also this implies that an empty model for service reception with random effect is better than an empty model for service reception without random effect.

### 3.3. Random intercept and fixed effect model

In the random intercept and fixed slope model, covariates are included but none of them are allowed to have a cluster-specific effect upon the response, i.e., each covariate's effect is assumed to be the same in the clusters. The probability of receiving service is allowed to vary across regions while level one covariates including in the fixed intercept are fixed or constant across regions. The results of the two-level random intercept and fixed slope model are presented in the following Table 3.

**Table 3:** The random intercept with fixed slope model.

Variables	Covariates	Estimate (S.E)	[95% Conf. int]	*p*-value
	Intercept	−2.1975 (0.2205)	(2.6297, −1.7653)	0.0001*
Age	20–34	−0.3177 (0.1383)	(−0.5888, −0.0466)	0.02161*
	35–49	−0.1653 (0.1590)	(−0.4769, 0.1463)	0.29861
Residence	Urban	1.5773 (0.0864)	(1.4080, 1.7466)	0.0001*
Religion	Protestant	−0.4348 (0.1110)	(−0.6524, −0.2172)	0.0001*
	Muslim	0.0939 (0.0937)	(−0.0898, 0.2776)	0.31637
	Others	−0.8208 (0.3127)	(−1.4435, −0.1981)	0.00867*
Woman education	Primary	0.3964 (0.0678)	(0.2635, 0.5293)	0.0001*
	Secondary	0.8606 (0.1328)	(0.6003, 1.1209)	0.0001*
	Higher	1.1515 (0.2446)	(0.6721, 1.6309)	0.0001*
Husband education	Primary	−0.0066 (0.0659)	(−0.1952, 0.0632)	0.92025
	Secondary	0.4669 (0.1252)	(0.2215, 0.7123)	0.00019*
	Higher	0.4807 (0.1029)	(0.2790, 0.6824)	0.0001*
Wealth	Middle	0.5043 (0.0794)	(0.3487, 0.6599)	0.0001*
	Rich	0.6626 (0.0736)	(0.5138, 0.8069)	0.0001*
Media exposure	Less than	0.1067 (0.0685)	(−0.0216, 0.2410)	0.11933
	At least	0.2914 (0.1080)	(0.0797, 0.5031)	0.00697*
	Almost	1.2456 (0.3798)	(0.5012, 1.9900)	0.00104*
Birth order	2–4	−0.5095 (0.0775)	(−0.6614, −0.3576)	0.0001*
	5 and above	−0.6331 (0.0938)	(−0.8169, −0.4493)	0.0001*
Contraceptive type	Yes	0.3737 (0.0643)	(0.2477, 0.4997)	0.0001*
ANC visit	1–3	1.4022 (0.0782)	(1.2489, 1.5555)	0.0001*
	4 and above	2.0173 (0.0790)	(1.8625, 2.1721)	0.0001*

**Table:** 

	Parameter	S.E.	[95% Conf. interval]
var(cons)	0.242	0.0814	(0.0825, 0.4015)
ICC	0.106		
Model selection			
Deviance	8,5118511	BIC	8,7338733
AIC	8,5278527	Deviance residual	1,5981598

*Significant variable.

According to the output of Table 3, the deviance-based chi-square is 1,598 (11,707–8,511) where 11,707 and 8,511 are the deviance of the two models as shown in Tables 2, 3 respectively. And this value is compared to Wald chi-square (22) = 1,598 with *p* = 0.000 which indicates that the random intercept model with the fixed slope is found to give a better t as compared to the random intercept model only. Moreover based on the AIC and BIC values for the fixed slope model with random intercept (AIC = 8,527, BIC = 8,733) are less than those for the random intercept model only (AIC = 11,711, BIC = 11,726). This concludes that the fixed slope model with random intercept was a better t as compared to the empty model with random intercept model. And it shows that the inclusion of level one covariates decreased regional variations from 1.82 (level-two variance without covariates) to 0.242, it indicates that there is a significant variation between regions in the probability of receiving delivery care service.

### 3.4. Random intercept model with random coefficients

In the random intercept model with random coefficients the women's level covariates are allowed to vary randomly across regions. In this section, the researcher investigates whether level-one covariates have random or fixed effects across regions. All variables included in the random intercept model are included in the random coefficient model. Estimates of this model show that the random slope variances of all included variables except place of residence and woman's educational level are zero, whereas the effect of other variables is the same for each region. The results of the random coefficient model are presented in Table 4.

**Table 4:** Parameter estimates of random coefficient model.

Variables	Covariates	Estimate (S.E)	[95% Conf. int]	*p*-value	OR
	Intercept	−2.2665 (0.2199)	(−2.6975, −1.8354)	0.0001*	–
Age	20–34	−0.3471 (0.1383)	(−0.6181, −0.076032)	0.0121*	0.707
	35–49	−0.2047 (0.1592)	(−0.5167, 0.10733)	0.1985	0.7808
Residence	Urban	1.7663 (0.1599)	(1.45289, 2.0797)	0.0001*	5.849
Religion	Protestant	−0.4136 (0.1125)	(−0.6228, −0.2042)	0.0002*	0.661
	Muslim	−0.7889 (0.3229)	(−1.38949, −0.1883)	0.0146*	0.4543
	Others	0.0647 (0.0948)	(−0.1211, 0.2505)	0.4946	1.0668
Womedu	Primary	0.4020 (0.0774)	(0.25029, 0.5537)	0.0001*	1.495
	Secondary	0.9074 (0.1537)	(0.6061, 1.2086)	0.0001*	2.478
	Higher	1.2481 (0.2777)	(0.7038, 1.7924)	0.0001*	3.484
Husedu	Primary	−0.0067 (0.0663)	(−0.1366, 0.1232)	0.9191	0.993
	Secondary	0.4666 (0.1033)	(0.2641, 0.6691)	0.0005*	1.595
	Higher	0.5267 (0.1037)	(0.3227, 0.72925)	0.0001*	1.9335
Wealth	Middle	0.5095 (0.0803)	(0.3521, 0.6668)	0.0001*	1.665
	Rich	0.6916 (0.0748)	(0.5449, 0.8382)	0.0001*	1.997
Mediaexp	Less than	0.0954 (0.0689)	(−0.0396, 0.2304)	0.1664	1.100
	At least	0.2397 (0.1097)	(0.0247, 0.4547)	0.0291*	1.271
	Almost	1.1211 (0.3803)	(0.3757, 1.8664)	0.0032*	3.068
Bordor	2–4	−0.5043 (0.0779)	(−0.6569, −0.3516)	0.0001*	0.604
	5+	−0.6110 (0.0941)	(−0.7954, −0.4265)	0.0001*	0.543
Contype	Yes	0.3580 (0.0651)	(0.2304, 0.4855)	0.0001*	1.4584
ANCvisit	1–3	1.4114 (0.0785)	(1.2654, 1.5574)	0.0001*	4.102
	4+	2.0247 (0.0794)	(1.8691, 2.1803)	0.0001*	7.574

**Table:** 

Random part			
	Parameter	S.E.	[95% Conf. interval]
var(constant)	0.2228	0.1086	0.0099, 0.4357
var(residence)	0.1634	0.0475	0.0703, 0.2565
var(womanedu)	0.0132	0.0236	0.00233, 0.0236
ICC	0.217		
cov(constant, residence)	–0.0763	0.0024	–0.0812, –0.0715
cov(cons, womanedu)	–0.0185	0.0183	–0.0543, 0.0174
cov(residence, womanedu)	0.0465	0.0084	0.0299, 0.0629
ICC	0.215		

*Significant variable.

In Table 4, the fixed part with two random covariates (place of residence and woman educational level) of the random coefficient model. From this output the estimated variance of intercept, slope of place of residence, and slope of woman's education were 0.2228, 0.1633, and 0.0132 respectively. The effect of intercept on region j is estimated to be −2.26655 + *U*_0*j*_ with a variance of 0.2228. The intercept variance of 0.2228 is interpreted as the between-region variance when all other variables are held constant (i.e., equal to zero). The between-region variance (individual region slopes) of place of residence and woman's education are estimated to be 0.1633 and 0.0132 respectively. So, there is a significant variation in the effect of place of residence and woman's educational level across regions in Ethiopia.

In addition to the variance of slope, it is important to interpret the covariance of random intercept and slope. Positive covariance between intercept and slopes implies that regions with higher intercepts tend to have on average higher slopes on the corresponding predictors. In other words, if the covariance of the random intercept and slope is a positive relationship, then as the random intercept increases, the random slope will increase. Therefore from Table 4, the covariance of the random intercept and slope of residence is a negative relationship, which means that as the random intercept increases, the random slope of place of residence decreases. This means that women living in urban are less likely not to deliver without the help of a health professional, and a similar interpretation for the slope of woman's educational level.

The results in Table 4 show that the inclusion of level one covariates, place of residence and woman's educational level, varying across regions significantly improved the random intercept model. The deviance-based Chi-square (deviance = 13.5) with degrees of freedom d.f = 5 was significant which indicates that the random coefficient model is a better fit as compared to the random intercept and fixed effect model. So, this concluded the random slope model was the best fit for this data.

### 3.5. Parameter interpretation of random coefficient model

In multi-level analysis, parameter interpretation is based on specific subjects or clusters. The parameter interpretation is conditional on the random effects, which is common for all individual women in the same cluster. Given the same random effects *b_j_*, the estimated odds ratio of delivery care service for the age group 20–34 is exp(−0.34703) = 0.707 times the odds of mothers in the age group 15–19 in the same *j*^th^ cluster, keeping constant the other fixed effect variables in the model. This implies that the estimated odds of receiving delivery care were 29.3% lower for mothers in the age group 20–34, compared to mothers in the age group 15–19, and mothers in urban areas were 484.9% (OR = 5.849) times more likely to receive delivery service compared to those in rural areas, controlling for other variables in the model and random effects at level two.

In the model, mothers who had primary level education had estimated odds of receiving service that were 49.5% (OR = 1.495) times higher than non-educated mothers, and the estimated odds for mothers with secondary and higher education were 147.8% (OR = 2.478) and 248.4% (OR = 3.484) times higher respectively. This indicates that mothers with secondary and higher education were 2 and 3 times more likely to receive delivery service, controlling for other variables in the model and random effects at level two. Mothers in middle-income households were 66.5% (OR = 1.665) more likely to receive delivery service than mothers in poor households. The estimated odds for mothers in rich households were 99.7% (OR = 1.997 times more likely to receive delivery service than mothers who live in poor households, after controlling for other variables in the model and random effects at level two. This indicates that as the wealth index of the household increases, the odds of receiving delivery service were also increased.

There is a significant difference between mothers who follow different types of media exposure. From the output, the estimated odds of receiving delivery service for mothers who follow media at least once a week was 27.1% (OR = 1.271) higher than mothers who didn't follow any media, and the estimated odds of mothers who follow media almost every day was 206.8% (OR = 3.068) higher than mothers without exposure to any mass media, after controlling for other variables in the model and random effects at level two.

At the given constant random effect, the odds of delivery care service utilization of mothers for birth order of 2–4 was 39.6% (OR = 0.604) lower than mothers with first birth order, and mothers with birth order 5 or higher were 45.7% (OR = 0.543) less likely to receive delivery service than mothers with first birth order at the fixed covariates and with the same random effects. Finally, mothers with 1–3 ANC visits were 310.2% (OR = 4.102) more likely to deliver with assistance of health professionals compared to mothers without ANC, and mothers with 4 or more ANC visits were 657.4% (OR = 7.574) times more likely to receive delivery service compared to mothers without ANC, after controlling for other variables in the model and random effects at level two. This indicates that there is a positive relationship between assistance during delivery and ANC visits.

## 4. Discussion

The aim of this study was to identify significant factors of delivery service in Ethiopia based on the 2016 EDHS data by using a multilevel model approach. The random slope of the multilevel logistic regression model showed that age, residence, religion, mother's educational level, partner's educational level, wealth index, exposure to mass media, birth orders, contraceptive use, and ANC visits were found significantly associated with delivery service utilization of mothers. The findings of this study were compared to other literature in this area.

Maternal age was associated with delivery care service utilization. Younger women were more likely to receive delivery care service than older women. This finding is consistent with the literature: mothers aged 15–19 and mothers aged 20–34 were more likely to receive delivery care service than mothers aged 34–49 (6, 7). This might be because older women consider that delivering without the aid of health professionals is not risky as they have experience in delivery without assistance. This study found that women living in urban areas were more likely to access delivery care service than rural women. This conclusion is similar to the results in the literature (8–10). The possible explanation for this could be that health and education infrastructures are highly concentrated in urban areas.

This study confirmed a significant positive association between the educational level of the mothers and the use of skilled assistance during delivery. Women with secondary education and above were significantly more likely to deliver at a health institution compared to women with primary education or below. The findings are similar to this study; the multiple logistic regression analysis showed that mothers who had not been formally educated were less likely to give birth at a health institution than those with secondary education and above (11). Education might help to get awareness and increase communication skills on health issues, including delivery.

The findings from this study indicate that the economic status of households had an association with access to delivery care service. Women in households with high wealth index were more likely to use delivery services than those with middle and low wealth index. This finding is consistent with studies conducted in Nigeria: women from wealthy households are more likely to utilize maternal health services than those from poor and middle income households (12). Another study in sub-Saharan Africa has also confirmed that women with the least wealth were least likely to use delivery services (3). A reason for this finding may be that the family members in households with higher economic status are more aware of accessible modern healthcare services and can afford those services easily. The costs of seeking skilled assistance at delivery may act as an important barrier to mothers from poor households.

Exposure to media was also a significant predictor of health facility delivery services. Mothers exposed to any mass media were more likely to use delivery services from health professionals during delivery than mothers without exposure, which is supported by (13). This finding is also similar to a study done in Nigeria which reported that mothers residing in communities with a higher proportion of exposure to media had higher odds of using health facility delivery services (12). Increased media exposure might help to increase the discussion of maternal issues within the community.

Like several studies, this study also found a negative association between birth order and the use of skilled delivery assistance during delivery. Women who had a large family, high birth order, and many children were less likely to deliver in a health facility, and this is consistent with most of the research conducted in sub-Saharan Africa but contradicts with the finding that households with large family size were more likely to deliver in a health facility (11, 14). This might be because large families help to look after small children when their mothers go to the health facility for delivery, and care for their property at home.

This study identified that mothers who use modern contraceptives were more likely to deliver with skilled assistance. This finding is supported by Buta: women who use modern contraceptives were three times more likely to deliver in a health facility compared to those who did not use contraceptives (8). Using modern contraceptives for family planning is important to build good awareness of and positive attitudes toward making connections between mothers and health facility materials, which is important to make a good connection and to deliver at a health facility with the aid of professionals.

The findings of this study revealed that women who receive less antenatal care had a higher likelihood of not delivering in a health facility with the assistance of professionals. A study in Nigeria concludes that the role of antenatal care has been emphasized (12). The finding is similar to the results of previous studies done in other countries like Ghana, Kenya, and others (15–17) and all these studies suggest that ANC has a significant effect on assistance during delivery.

Although from the previous studies the occupation of mothers and the sex of the head of household were significantly associated with delivery care service utilization, in this study these covariates didn't become significant determinant factors for the use of delivery care service.

## 5. Conclusion

Among the total mothers in the study, the proportion of coverage of delivery care service utilization was merely 33.3%. The random coefficient model with two random slopes was the best model for this data and from this model age, place of residence, religion, woman and partners' educational level, wealth index of household, media exposure, birth order number, contraceptive use, and number of ANC visits were statistically significantly related to delivery care service utilization. The study revealed that there was a between and within regional variation in delivery care service, and the multi-level model identified that the effect of place of residence and woman's educational level varied across regions whereas the effect of other covariates was similar.

## Data Availability

The original contributions presented in the study are included in the article, further inquiries can be directed to the corresponding author.
